# Paraoxonase and arylesterase activity of paraoxonase 1 and oxidative stress parameters in cervical intraepithelial neoplasia

**DOI:** 10.11613/BM.2024.010701

**Published:** 2023-12-15

**Authors:** Dražan Butorac, Ivana Ćelap, Sanja Kačkov Maslać, Tomislav Miletić, Andrea Hulina Tomašković, Petra Turčić, Dubravka Rašić, Ivana Stojanović, Marija Grdić Rajković

**Affiliations:** 1 Clinic of Gynecology and Obstetrics, Sestre milosrdnice University Hospital Center, Zagreb, Croatia; 2 Department of Clinical Chemistry, Sestre milosrdnice University Hospital Center, Zagreb, Croatia; 3 Medical biochemistry laboratory, Polyclinic Bonifarm, Zagreb, Croatia; 4 Polyclinic Aviva, Zagreb, Croatia; 5 Department of Medical biochemistry and Hematology, Faculty of Pharmacy and Biochemistry, University of Zagreb, Zagreb, Croatia; 6 Department of Pharmacology, Faculty of Pharmacy and Biochemistry, University of Zagreb, Zagreb, Croatia; 7 Unit of Toxicology, Institute for Medical Research and Occupational Health, Zagreb, Croatia

**Keywords:** paraoxonase 1, cervical intraepithelial neoplasia, oxidative stress

## Abstract

**Introduction:**

Paraoxonase 1 (PON1) is the enzyme that removes carcinogenic radicals from lipids. The aim of the study was to investigate the differences in PON1 activity and oxidation stress parameters between patients with cervical intraepithelial neoplasia (CIN) and healthy controls.

**Materials and methods:**

The study included 65 women with CIN and 109 healthy women. Lipid parameters were determined on Cobas Integra 400 plus (Roche, Mannheim, Germany). Tiols and reduced glutathione (GSH) were determined spectrophotometric using Eliman reagent. Activity of PON1 was assessed with two substrates, paraoxon and phenylacetate by spectrophotometric method. Malondialdehyde (MDA) was determined by high performance liquid chromatography (Shimadzu Corporation, Kyoto, Japan). Mann-Whitney-test, t-test, χ2-test, correlation and logistic regression was used in statistical analysis. P < 0.05 was considered statistically significant.

**Results:**

The basal (P = 0.929) and NaCl-stimulated (P = 0.985) PON1 activity and activities standardised on the concentration of high-density lipoprotein (HDL; P = 0.076; P = 0.065, respectively) and apolipoprotein AI (apo AI; P = 0.444; P = 0.499, respectively) as well as PON1 phenotypes (P = 0.842) did not differ significantly between the groups. The PON1 arylesterase activity (53±19 kU/L vs. 77±17 kU/L; P < 0.001) and HDL-standardized activity (37 (28-44) kU/mmol *vs*. 43 (37-50) kU/mmol; P < 0.001) and apoAI (29±11 kU/g *vs*. 44±11 kU/g; P < 0.001) was significantly reduced in the CIN group. The concentration of the thiol groups was similar (P = 0.519), of MDA was lower (0.39 (0.27-0.55) µmol/L *vs*. 0.76 (0.57-1.15) µmol/L; P < 0.001) and of GSH was higher (112.0 (66.0-129.6) µg/mL *vs*. 53.4 (34.8-134.4) µg/mL; P < 0.001) in the CIN group.

**Conclusion:**

Reduced PON1 arylesterase activity, lower MDA and higher GSH concentration were observed in CIN patients.

## Teaser

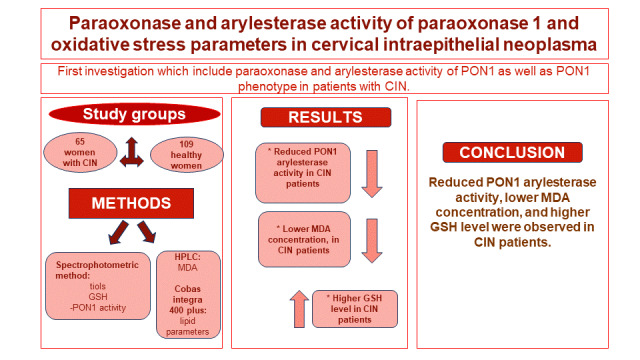

## Highlights

Reduced paraoxonase 1 arylesterase activity was observed in cervical intraepithelial neoplasia

Malondialdehyde was lower in the cervical intraepithelial neoplasia group

Reduced glutathione was higher in cervical intraepithelial neoplasia

## Introduction

Cervical intraepithelial neoplasia (CIN) is characterized by different degrees of dysplasia of the squamous cells in the cervical epithelium. Thus, CIN1 is characterized by mild, CIN2 by moderate, and CIN3 or carcinoma *in situ* by severe dysplasia. Cervical intraepithelial neoplasia can progress to carcinoma *in situ* and invasive cervical carcinoma over time if it is not treated at an early stage. Cervical cancer is the fourth most common malignant tumour among women. Its main etiologic factor in carcinogenesis is human papilloma virus (HPV) infection (*1*, *2*). It seems that the presence of HPV is essential for cervical cancer, but other cofactors are also important, and they include oral contraceptives uptake, low social status, malnutrition, cigarette smoking, lower age of first sexual intercourse and number of sexual partners. One of the cofactors that attracted the attention of researchers is oxidative stress (*2*, *3*). It is well known that oxidative stress and reactive oxygen species (ROS) are involved in the initiation and promotion of cancer development and research have shown that lipid peroxidation can play a major role in cancerogenesis (*4*-*6*). A high concentration of ROS or its insufficient removal by cell or plasma antioxidants will result in low-density lipoprotein (LDL) peroxidation. Oxidized LDL (oLDL) is considered an independent mitogen factor that induces cell proliferation or death and can contribute to the development and progression of cancer by increasing of cytokines and growth factors release (*6*). Paraoxonase 1 (PON1) has antiatherogenic and antioxidative effects, it prevents high-density lipoprotein (HDL) and LDL oxidation, so it is reasonable to assume that this enzyme, through its antioxidant activity, has influence on carcinogenesis as well as on the progression of premalignant lesion to invasive cervical carcinoma. In various pathological conditions related to oxidative stress, including cardiovascular disease, diabetes mellitus, and patients who require haemodialysis, a decrease in PON 1 activity has been found (*7*). Activity of PON1 is also reduced in various types of cancer (*8*-*14*). For example, PON1 paraoxonase and arylesterase activity is reduced in patients with ovarian cancer and is related to cancer stage, histological type, and the concentration of the tumour marker CA-125 (*10*). Furthermore, a decrease in PON1 paraoxonase and arylesterase activity has been established in patients with endometrial and breast cancer (*8*, *9*, *14*). Samra *et al.* determined the PON1 paraoxonase and arylesterase activity and serum PON1 concentrations in patients with different types of cancer and found its decreased values in breast, prostate, lung cancer, and non-Hodgkin lymphoma (*12*). This research is the only one where the PON1 paraoxonase and arylesterase activity and the PON1 concentration were investigated in patients with cervical cancer, and where its decreased activity together with its lowered concentration was found. Available literature data did not reveal any scientific paper in which the paraoxonase or arylesterase activity of PON1 in CIN patients was determined.

We hypothesized that the patients with CIN have elevated markers of oxidative stress, as well as decreased activity of the antioxidase enzyme PON1 compared to the healthy women. The aim of this study was to determine whether there are differences in the markers of oxidative stress and PON1 enzyme activity between CIN patients and healthy controls.

## Materials and methods

### Study design

This case-control study was conducted in Department of Gynaecology and Obstetrics and the Department of Clinical Chemistry, Sestre milosrdnice University Hospital Centre, Zagreb, Department of Medical Biochemistry and Hematology, Faculty of Pharmacy and Biochemistry, University of Zagreb, Policlinic Aviva, Zagreb and Polyclinic Bonifarm, Zagreb. Patients with CIN were recruited during December 2013 to December 2014 in the Department of Gynaecology and Obstetrics, Sestre milosrdnice University Hospital Centre in Zagreb. Healthy patients were recruited during systematic examination, at the same time period in Polyclinic Aviva. Sample analyses were conducted in the Department of Clinical Chemistry, Sestre milosrdnice University Hospital Centre in Zagreb, Department of Medical Biochemistry and Haematology, Faculty of Pharmacy and Biochemistry, University of Zagreb and Polyclinic Bonifarm, Zagreb.

### Subjects

The study included 65 women with diagnosed CIN. Cytological screening (Pap smear) and classification according to the pattern „Zagreb 2002” were performed followed by colposcopy-directed biopsy and pathohistological analysis (PHD) (*15*). From 65 patients with pathohistologically confirmed CIN, 18 were classified as CIN I, 23 as CIN II, and 24 as CIN III, with respect to 18 patients with low grade lesion (CIN I) and 47 with high grade lesions (CIN II, CIN III and carcinoma *in situ*) according to The Bethesda classification (*16*). The inclusion criteria for the patient group was pathohistological confirmation of CIN diagnosis. Exclusion criteria were the presence of renal or liver disease, hypertension, myocardial infarction, cerebrovascular insult, pectoral angina, diabetes mellitus, polycystic ovary syndrome, endometriosis, malignant disease, pregnancy as well as medications that alter fat and carbohydrate metabolism.

The control group included 109 healthy women matched by age and lifestyle habits with CIN patients. All women were examined by a general practitioner and gynaecologist and had a normal Pap smear in the period of the past two years. The exclusion criteria were the same as for the patients group.

The study was approved by the Ethics Committee of Clinical Hospital Centre Sestre milosrdnice, Zagreb, Croatia (EP-7802/12-2) and the Polyclinic Aviva, Zagreb, Croatia. All participants signed an informed consent.

### Samples

Serum samples were collected in tubes with clot activator after 12 hours of fasting (Greiner Bio-One, Kremsmünster, Austria). After 30 minutes of spontaneous clotting, the samples were centrifuged at 2000xg for 10 minutes at room temperature and the sera were stored at - 20 °C until further analysis. For DNA isolation, EDTA blood samples (Greiner Bio-One, Kremsmünster, Austria) were collected and stored at - 20 °C until further analysis.

### Methods

The concentrations of the lipid parameters (triglyceride, total cholesterol, HDL cholesterol, LDL cholesterol, apolipoprotein AI (apoAI) and apolipoprotein B (apoB)) were determined with the original reagents, according to the manufacturer’s protocol on Cobas Integra 400 plus (Roche, Mannheim, Germany).

The activity of PON1 in serum was assessed by using two different substrates: paraoxon (PON1 paraoxonase activity; Sigma Aldrich Chemie GmbH, Darmstadt, Germany) and phenylacetate (PON1 arylesterase activity; Sigma Aldrich Chemie GmbH, Darmstadt, Germany) according to the protocol described below. The PON1 paraoxonase activity was measured without (basal PON1 paraoxonase activity; POX) and with NaCl (salt stimulated PON1 paraoxonase activity; POX1). Activities of POX, POX1 and PON1 arylesterase (ARE) were standardized on the concentration of HDL (POX/HDL, POX1/HDL, ARE/HDL, respectively) and ApoAI (POX/ApoAI, POX1/ApoAI, ARE/apoAI, respectively) (*17*, *18*).

The PON1 phenotype was determined using the double substrate method as previously described (*17*).

The concentration of tiol groups was determined by using Ellman reagent (5,5’-dithio-bis-2-nitrobenzoic acids or DTNB; Sigma Aldrich Chemie GmbH, Darmstadt, Germany) by the method described by Hu (*19*).

For measuring the concentration of malondialdehyde (MDA) the modified method was used (*20*, *21*). The method is based on the formation of a red colour product in reaction of MDA with thiobarbituric acid (TBA) in the presence of antioxidant butylated hydroxytholuene (BHT). Colour products are determined by high performance liquid chromatography (HPLC; Shimadzu Corporation, Kyoto, Japan). Briefly, reaction mixture contained 50 µL serum, 5 µL 0.2% BHT (Sigma Aldrich Chemie GmbH, Germany), 750 µL 1% H_3_PO_4_ (Kemika, Zagreb, Croatia), 250 µL 0,6% TBA (Sigma Aldrich Chemie GmbH, Darmstadt, Germany) and 445 µL H_2_O (Merck, Germany). The reaction mixture was incubated 20 minutes at 100°C and stopped by placing reaction mixture in an ice bath. The mobile phase (pH 6.8) for HPLC contained 50 mmol/L KH_2_PO_4_ (Merck, Germany) and methanol (60:40; Kemika, Zagreb, Croatia). The mobile phase flow rate was adjusted to 1 mL/min. The reverse phase column C18 (Merck, Darmstadt, Germany) empire at 32 °C was used during the assay. The wavelength of the UV detector was 532 nm, and the retention time of MDA on the analytical column was 2.5 minutes. The concentration of MDA in the samples was determined using the standard curve of standard 1,1,3,3-tetramethoxy propane (Merck, Darmstadt, Germany).

The reduced glutathione concentration was determined by the spectrophotometric method of Ellman which is based on the binding of 5,5’-dithiobis-2-nitrobenzoic acid or DTNB (*22*). Briefly, the reaction mixture contained 50 μL DTNB (Sigma Aldrich Chemie GmbH, Darmstadt, Germany), 850 μL phosphate buffer, and 100 μL sample serum, or 100 μL reduced glutathione (GSH) standard (Merck, Darmstadt, Germany) or 100 μL water for blank. Absorbance was measured at 410 nm (Cecil Aquarius CE 7200, Cecil Instruments Limited, Cambridge, United Kingdom) and the concentration of reduced GSH is determined using the standard curve.

### Statistical analysis

Data are presented as median and interquartile range, mean and standard deviation or as proportions. The Kolmogorov-Smirnov test was used to test the assumption of normal distribution. Quantitative data was analysed using the t-test or Mann-Whitney rank sum test. Chi-square test was used for comparison of proportions. To test the correlation between ARE and HDL, as well as between ARE and apoAI, Spearman or Pearson correlation test was used. Logistic regression was used to determine the strength of association between tested markers and CIN. P values less than 0.05 were considered statistically significant. Statistical analysis was performed using SigmaStat for Windows, version 3.0 (2003. SPSS Inc, Erkrath, Germany) and MedCalc statistical software ver.14.8.1.0. (MedCalc Software Lcd., Ostend, Belgium).

## Results

The concentrations of the lipid parameters are given in Table 1. Concentration of HDL cholesterol was significantly lower (1.4 (1.3-1.6) mmol/L *vs.* 1.8 (1.5-2.0) mmol/L, P < 0.001)) while the concentration of apoAI (1.88 (1.74-2.07) g/L *vs.* 1.73 (1.58-1.99) g/L, P = 0.006)) was significantly higher in patients with CIN.

**Table 1:** Concentrations of lipids, oxidative stress markers and PON1 paraoxonase and arylesterase activity

	**Control group** **(N = 109)**	**CIN group** **(N = 65)**	**P**
Triglyceride (mmol/L)	1.0 (0.7-1.3)	0.9 (0.7-1.2)	0.716
Cholesterol (mmol/L)	5.3 (4.9-6.4)	5.3 (4.9-6.1)	0.562
HDL (mmol/L)	1.8 (1.5-2.0)	1.4 (1.3-1.6)	< 0.001
LDL (mmol/L)	3.1 (2.7-3.8)	3.3 (2.8-4.1)	0.228
apoAI (g/L)	1.73 (1.58-1.99)	1.88 (1.74-2.07)	0.006
apoB (g/L)	0.93±0.26	0.88±0.25	0.204
Thiols (mmol/L)	0.36 (0.33-0.39)	0.35 (0.30-0.40)	0.519
MDA (µmol/L)	0.76 (0.57-1.15)	0.39 (0.27-0.55)	< 0.001
GSH (µg/mL)	53.4 (34.8-134.4)	112.0 (66.0-129.6)	< 0.001
POX (U/L)	96 (67-242)	107 (69-209)	0.929
POX/HDL (U/mmol)	51 (39-134)	73 (49-153)	0.076
POX/apoAI (U/g)	54 (37-137)	51 (40-115)	0.444
POX1 (U/L)	194 (131-426)	199 (136-366)	0.985
POX1/HDL (U/mmol)	101 (75-237)	133 (97-271)	0.065
POX1/apoAI (U/g)	105 (74-245)	101 (78-207)	0.499
ARE (kU/L)	77±17	53±19	< 0.001
ARE/HDL (kU/mmol)	43 (37-50)	37 (28-44)	< 0.001
ARE/apoAI (kU/g)	44±11	29±11	< 0.001
Data are shown as median (interquartile range) or as mean ± standard deviation. The difference between groups was tested by Mann-Whitney test or t-test. P < 0.05 was considered statistically significant. CIN - cervical intraepithelial neoplasia. HDL - high density lipoproteine. LDL - low density lipoprotein. apoAI - apolipoprotein AI. apoB - apolipoprotein B. MDA - malondialdehyde. GSH - reduced glutathione. PON1 - paraoxonase 1. POX - basal PON1 paraoxonase activity. POX1 - salt stimulated PON1 paraoxonase activity. ARE - PON1 arylesterase activity. POX/HDL - basal PON1 paraoxonase activity standardized on the concentration of HDL. POX/apoAI - basal PON1 paraoxonase activity standardized on the concentration of apoAI. POX1/HDL - salt stimulated PON1 paraoxonase activity standardized on the concentration of HDL. POX1/apoAI - salt stimulated PON1 paraoxonase activity standardized on the concentration of apoAI. ARE/HDL - PON1 arylesterase activity standardized on the concentration of HDL. ARE/apoAI - PON1 arylesterase activity standardized on the concentration of apoAI.			

The concentration of free thiols, MDA and GSH as markers of oxidative stress are also given in Table 1. The concentration of MDA was lower (0.39 (0.27-0.55) µmol/L *vs.* 0.76 (0.57-1.15) µmol/L, P < 0.001)) while GSH was higher (112.0 (66.0-129.6) µg/mL *vs.* 53.4 (34.8-134.4) µg/mL, P < 0.001)) in patients with CIN.

Further, in Table 1 are presented results of the PON1 paraoxonase and arylesterase activity as well as those activities standardized on the concentration of HDL and apoAI. We observed significantly lower ARE (53±19 kU/L *vs.* 77±17 kU/L, P < 0.001], ARE/HDL [37 (28-44) kU/mmol *vs.* 43 (37-50) kU/mmol, P < 0.001)) and ARE/apoAI (29±11 kU/g *vs.* 44±11 kU/g, P < 0.001) in patients with CIN. Compared to the control group in the group of patients, ARE was lower by 31%, ARE/HDL by 14%, and ARE/apoAI by 34%. Although statistically significant, there was no correlation between ARE and HDL and ARE and apoAI in control group (r_p_ = 0.32; *P* < 0.001 and r_s_ = 0.25; *P* = 0.009). Furthermore, no correlation was found between ARE and HDL nor ARE and apoAI in CIN group of patients (r_s_ = 0.08; *P* = 0.546 and r_s_ = 0.08; *P* = 0.533).

The results of PON1 phenotype analysis are given in Table 2. No different phenotype distribution was observed between the study groups.

**Table 2:** PON1 phenotype distribution

	**AA phenotype** **N (proportion)**	**AB phenotype** **N (proportion)**	**BB phenotype** **N (proportion)**	**P**
Control group (N = 109)	62 (0.57)	35 (0.32)	12 (0.11)	0.842
CIN group (N = 65)	34 (0.52)	23 (0.36)	8 (0.12)	
Results are shown as number (N) and proportion of the examinees with certain phenotype. P value was obtained using χ^2^ -test. P < 0.05 was considered statistically significant. PON1 - paraoxonase 1. CIN - cervical intraepithelial neoplasia. AA phenotype - low activity homozygotes. AB phenotype - middle range activity heterozygotes. BB phenotype - high activity homozygotes.				

Multivariate logistic regression analysis has shown statistically significant associations between ARE/HDL (1.31 (1.18-1.46); P < 0.001), ARE/apoAI (0.66 (0.58-0.75); P < 0.001) and MDA ((0.06 (0.02-0.24); P < 0.001) and CIN (Table 3). Although statistically significant, obtained odds ratio (OR) for GSH ((1.01 (1.00-1.03); P = 0.024) is very close to 1 and probably does not contribute to prediction of CIN status.

**Table 3:** Multivariate logistic regression of CIN status according to measured biochemical parameters.

	**Univariate** **regression analysis**		**Multivariate** **regression analysis**	
	**OR (95% CI)**	**P**	**OR (95% CI)**	**P**
HDL (mmol/L)	0.09 (0.03-0.25)	< 0.001		
apoAI (g/L)	4.06 (1.40-11.76)	0.008		
ARE (kU/L)	0.93 (0.91-0.96)	< 0.001		
ARE/HDL (kU/mmol)	0.96 (0.93-0.98)	< 0.001	1.31 (1.18-1.46)	< 0.001
ARE/apoAI (kU/g)	0.87 (0.84-0.91)	< 0.001	0.66 (0.58-0.75)	< 0.001
MDA (µmol/L)	0.10 (0.04-0.29)	< 0.001	0.06 (0.02-0.24)	< 0.001
GSH (µmol/L)	1.01 (1.00-1.02)	< 0.001	1.01 (1.00-1.03)	0.024
P < 0.05 was considered statistically significant. OR - odds ratio. CI - confidence interval. HDL - high density lipoproteine. apoAI - apolipoprotein AI. MDA - malondialdehyde. GSH - reduced glutathione. ARE - PON1 arylesterase activity. ARE/HDL - PON1 arylesterase activity standardized on the concentration of HDL. ARE/apoAI - PON1 arylesterase activity standardized on the concentration of apoAI.				

## Discussion

Our study showed reduced PON1 arylesterase activity as well as MDA concentration in CIN patients. On the other hand, the GSH value was significantly higher in these patients.

It is well known that oxidative stress plays an important role in the developing of different types of cancer, including cervical cancer. Researchers also showed the presence of excess ROS in CIN patients. Infection with HPV virus that incorporates into the cellular genome results with higher ROS due to the expression of oncoproteins and the reduction of circulating antioxidants such as superoxide dismutase, catalase, glutathione peroxidase and GSH (*5*, *23*-*26*). Paraoxonase 1 is one of the enzymes which possess antioxidant activity, and lower PON1 activity exposed subjects to higher oxidative stress. Paraoxonase 1 prevents the formation of oxidized LDL and inactivates LDL-derived oxidized phospholipids and also prevents oxidation of phospholipids in HDL. Its antioxidative role has been attributed to the neutralization of fatty acid hydroperoxide, cholesterol ester hydroperoxide and hydrogen peroxide. It is involved in the detoxification of carcinogenic, lipid-soluble free radicals product of lipid peroxidation (*9*, *11*, *27*).

Reduced PON1 activity was reported in different types of female cancer such as breast, ovarian, and endometrial cancer (*8*-*10*, *13*, *14*). However, limited research data are available on PON1 paraoxonase and arylesterase activity as well as PON1 concentration in patients with cervical cancer (*12*). Furthermore, PON1 has been implicated in the pathogenesis of different inflammatory diseases. It was shown that proinflammatory cytokines like IL-1β and TNFα, downregulate PON1 expression and secretion by liver cells, so it is presumed that lower concentration of PON1 in serum could be the result of a long-lasting inflammation (*11*). Infection with HPV results in the activation of the host immune system and, among other, the release of a wide range of cytokine like TNFα (*1*). Our results indicate that the reduction of the PON1 arylesterase activity is present in the premalignant phase of the disease and could be one of the factors which can lead to the development of cervical cancer in future.

The PON1 arylesterase activity serves to assess the concentration of PON1, as its distribution is not polymorphic as the PON1 paraoxonase activity (*28*). Obtained results indicate not only the decrease in PON1 arylesterase activity but also the possible decrease in sera PON1 concentration in patients with CIN.

Paraoxonase 1 is synthetized in the liver and secreted in the blood where is meanly associated to the HDL. High-density lipoprotein and apoAI are important to maintain normal serum PON1 activity (*7*). To exclude the influence of the changes in the concentrations of these two parameters, PON1 activity was standardized to HDL and apoAI concentrations. The results of lower ARE/HDL and ARE/apoAI in CIN group in comparison to controls indicated that lowered ARE in patients with CIN was not a result of observed changes in HDL and apoAI concentrations. Absence of correlation between HDL, apoAI and ARE in the control group and CIN group also supports our previous conclusion.

Phenotype of PON1 is a result of genetic factors or polymorphisms in the promotor or coding regions of the *PON1* gene, as well as the different non-genetic factors (*7*). We did not observe different phenotype distribution between the study groups. Available literature search did not provide any study on the PON1 phenotype in patients with CIN.

In addition to the reduced ARE, we observed significantly lower MDA concentration in CIN group compared to control group. On the other hand, GSH is higher in the patients with CIN compared to controls. According to obtained results, we could speculate that in premalignant phase of the disease different antioxidation mechanisms are activated to prevent oxidative stress in CIN patients. Our results of oxidative stress markers do not correspond to the data in the published literature (*4*, *29*, *30*). However, different dietary and lifestyle habits as well as environmental factors in different parts of the word could result in discrepant findings. Multivariate regression analysis has shown statistically significant association of ARE/HDL, ARE/apoAI and MDA and CIN.

The main limitation of our study is relatively small sample size and the absence of the longitudinal follow up.

To the best of our knowledge, this research was the first one which include paraoxonase and arylesterase activity of PON1 as well as PON1 phenotype in patients with CIN. The CIN patient has reduced PON1 arylesterase activity, lower MDA and higher GSH concentration compared to the control group.

Further cohort investigation on larger group of patients with CIN and determination of PON1 activity in subgroups of patients with different CIN stages are needed to reveal the significant importance of this enzyme in CIN.

## Data Availability

The data generated and analysed in the presented study are available from the corresponding author on request.
